# Editorial: Community series in the immunosuppressive tumor microenvironment and strategies to revert its immune regulatory milieu for cancer immunotherapy, volume II

**DOI:** 10.3389/fimmu.2025.1724894

**Published:** 2025-10-28

**Authors:** Mazdak Ganjalikhani Hakemi, Nair Jayakumar

**Affiliations:** ^1^ Regenerative and Restorative Medicine Research Center (REMER), Research Institute for Health Sciences and Technologies (SABITA), Istanbul Medipol University, Istanbul, Türkiye; ^2^ Women’s Malignancies Branch, National Cancer Institute (NCI), Bethesda, MD, United States

**Keywords:** cancer, immunotherapy, immunometabolism, immune regulation, immunosuppression, tumor microenvironment (TME)

The advent of cancer immunotherapy has transformed oncology, establishing immune-based strategies as essential pillars of modern cancer care. Yet, despite remarkable progress, durable clinical responses remain inconsistent, with many patients experiencing limited benefit or relapse. A major determinant of therapeutic efficacy is the tumor microenvironment (TME), a highly dynamic ecosystem of malignant, stromal, and immune cells, together with extracellular matrix components and soluble mediators. The TME frequently evolves into an immunosuppressive and pro-angiogenic milieu that fosters tumor adaptation and immune escape (1–3). Understanding and modulating the TME remain critical for unleashing the full potential of immunotherapy (4).

Building on the foundation of *Volume I*, this second volume of the community series *“The Immunosuppressive Tumor Microenvironment and Strategies to Revert its Immune Regulatory Milieu for Cancer Immunotherapy”* brings together original research, case studies, reviews, and systematic analyses that collectively address the multifaceted barriers imposed by the TME. The eleven accepted articles span rare cancer case insights, prognostic biomarkers, stromal dynamics, metabolic regulation, immune cell heterogeneity, and therapeutic innovations. Together, these contributions highlight the urgent need for sintegrative strategies that combine biomarker discovery, mechanistic insights, and translational approaches to overcome immunosuppression in cancer.

## Case-based and disease-specific insights

A key dimension of this Research Topic is the characterization of rare or poorly studied tumors, where limited therapeutic options underscore the value of immune-based approaches. Liu et al. reported a giant thoracodorsal liposarcoma illustrating the diagnostic and therapeutic challenges posed by rare soft-tissue malignancies. Histopathology revealed infiltration of M2 macrophages, with tumor cores showing a higher concentration of uncommitted M0 macrophages and regulatory T cells, highlighting the role of a dynamic immunosuppressive TME in supporting disease pathogenesis and therapy resistance and offering clinicopathological insights into potential immune-targeted strategies in such rare sarcomas.

In a related review, Zhao et al. discussed the roles of key immunological features that modulate the TME in high-grade endometrial stromal sarcoma (HGESS), a highly aggressive tumor with poor prognosis. They explored how dysfunctional, exhausted cytotoxic CD8^+^ T cells (CTLs), myeloid suppressor cells, and dendritic cells play essential roles in reshaping the TME to support tumor growth. They evaluated therapeutic strategies including oncolytic viruses, adoptive T-cell transfer, immune checkpoint inhibitors, and personalized mRNA vaccines, providing valuable insights into future approaches and translational gaps that need to be addressed to improve outcomes in these rare sarcomas.

## Stromal barriers and prognostic biomarkers

Essential roles of stromal architecture and immune checkpoints in shaping clinical outcomes. Were explored by Da et al. in bladder cancer, a high stroma–tumor ratio (STR). Their studies demonstrated strong correlation between immunosuppression, T-cell exhaustion, and poor prognosis. The predictive role of stromal content towards an immunosuppressive environment and the likelihood of response to a specific immunotherapy was the highlight of this study (5–7). Such associations were further demonstrated by Ma et al. in gastric cancer, where multi-omic analyses were used to identify an SPP1+/C1QC+ macrophage/exhausted CD8+ T cell axis that contributed to an immunosuppressive barrier. These stromal–immune interactions spatially orchestrate anti-tumor immunity suppression and provide molecular markers for stratification and therapy adaptation (5–7). The clinical relevance of such molecular markers was highlighted by Wu et al. who reported PD-1 and LAG-3 co-expression as strong predictors of survival in ovarian cancer, establishing these molecules as a rational combinatorial checkpoint target. Collectively, these studies underscore stromal indices and checkpoint co-expression as key determinants for guiding immunotherapy (8–10).

## Immunometabolic regulation of tumor progression

Metabolic dysregulation critically influences tumor immune evasion. In AML, Bao et al. demonstrated that hypercholesterolemia and CES1, a gene involved in lipid metabolism, are independent risk factors for CNS relapse. CES1, a potential therapeutic target, enhanced fatty acid oxidation, M2 macrophage polarization, and immune suppression, linking lipid metabolism to relapse.

Li et al. analyzed a decade of literature of lactate research in cancer highlighting its central role as a metabolic regulator of cancer immunity through metabolic–epigenetic crosstalk, lactate-mediated signaling, and immunemodulation of the TME. Emerging areas include metabolic intervention and drug delivery, supporting targeting metabolic pathways to reprogram the TME (11, 12).

## Functional heterogeneity of immune subsets

An understanding of the immune subset diversity or heterogeneity within the TME is critical for precision therapy. Jiang et al. profiled γδ T cells in AML, identifying subsets with distinct functions, such as the strong correlation between NKG2D+ TIGIT− Vδ1 cells and improved survival, supporting their potential as biomarkers and/or therapeutic candidates in adoptive transfer approaches (13, 14).

In their elegantly designed study, Wang et al. demonstrated how a heterogeneous immune landscape plays essential predictive roles in determining the outcome of targeted immune therapy in adenoid cystic carcinoma of the head and neck. Their studies demonstrated how a desired higher immune infiltration is often countered by increased dominance of suppressive macrophages, revealing the paradox of concurrent immune abundance and dysfunction. Together, these studies demonstrate the importance of dissecting immune heterogeneity towards designing context-specific interventions (13–15).

## Molecular drivers of immune escape and innovative therapeutics

In many cancers like multiple myeloma, an aggressive and incurable hematological malignancy, immune evasion and its regulatory molecular determinants are essential to their progression. Liu et al. demonstrated how DNp73, an inhibitor of TP53, induced proliferation, drug resistance, and immune evasion, by targeting MYCN and upregulating MYC target genes PD-L1 and CD47, highlighting DNp73 as a biomarker for immunotherapy targeting PDL1/CD47 blockade in patients (16).

Akimova et al. introduced a novel approach using antisense oligonucleotides to selectively target FOXP3+ regulatory T cells. This strategy depleted intratumoral Tregs without affecting peripheral populations, enhancing effector T-cell activity and inducing significant tumor regression in preclinical models. These findings provide proof-of-concept for precise targeting of immunosuppressive cells in the TME (1, 16).

## Outlook and future directions

The diverse contributions in this second volume collectively underscore the complexity of the immunosuppressive TME and the multiplicity of strategies required to counteract it. Several recurring themes emerge:

1. Stromal orchestration of immune suppression as a central barrier across multiple tumor types.
2. Immunometabolic reprogramming, particularly lipid and lactate metabolism, as key drivers of immune dysfunction.
3. Immune checkpoint combinations and functional subset profiling as critical tools for precision immunotherapy.
4. Novel therapeutic modalities, from antisense oligonucleotides to adoptive T-cell transfer, highlighting innovative avenues for clinical translation.

Future research should prioritize multi-omic integration, spatial transcriptomics, and longitudinal patient profiling to comprehensively map TME dynamics. Moreover, clinical translation will require carefully designed combinatorial regimens, biomarker-guided stratification, and robust preclinical validation to ensure safety and efficacy.

## Conclusion

This collection of articles in *Volume II of the Community Series on the Immunosuppressive Tumor Microenvironment* provide valuable insights into the complex multifactorial barriers to cancer immunotherapy and provides innovative strategies for reprogramming the TME. By integrating stromal biology, metabolic regulation, immune subset heterogeneity, and novel therapeutic approaches, the contributions collectively advance our understanding of how to modulate immunosuppressive networks. Ongoing collaborative efforts will be vital to translating these insights into effective clinical strategies, ultimately leading to better outcomes for patients with diverse malignancies.
